# Epidemiology and Immune Pathogenesis of Viral Sepsis

**DOI:** 10.3389/fimmu.2018.02147

**Published:** 2018-09-27

**Authors:** Gu-Lung Lin, Joseph P. McGinley, Simon B. Drysdale, Andrew J. Pollard

**Affiliations:** ^1^Oxford Vaccine Group, Department of Paediatrics, University of Oxford, Oxford, United Kingdom; ^2^National Institute for Health Research, Oxford Biomedical Research Centre, Oxford, United Kingdom; ^3^Department of Paediatrics, St George's University Hospitals NHS Foundation Trust, London, United Kingdom

**Keywords:** viral sepsis, epidemiology, immune pathogenesis, herpes simplex virus, human enterovirus, human parechovirus, influenza virus, dengue virus

## Abstract

Sepsis is a life-threatening organ dysfunction caused by a dysregulated host response to infection. Sepsis can be caused by a broad range of pathogens; however, bacterial infections represent the majority of sepsis cases. Up to 42% of sepsis presentations are culture negative, suggesting a non-bacterial cause. Despite this, diagnosis of viral sepsis remains very rare. Almost any virus can cause sepsis in vulnerable patients (e.g., neonates, infants, and other immunosuppressed groups). The prevalence of viral sepsis is not known, nor is there enough information to make an accurate estimate. The initial standard of care for all cases of sepsis, even those that are subsequently proven to be culture negative, is the immediate use of broad-spectrum antibiotics. In the absence of definite diagnostic criteria for viral sepsis, or at least to exclude bacterial sepsis, this inevitably leads to unnecessary antimicrobial use, with associated consequences for antimicrobial resistance, effects on the host microbiome and excess healthcare costs. It is important to understand non-bacterial causes of sepsis so that inappropriate treatment can be minimised, and appropriate treatments can be developed to improve outcomes. In this review, we summarise what is known about viral sepsis, its most common causes, and how the immune responses to severe viral infections can contribute to sepsis. We also discuss strategies to improve our understanding of viral sepsis, and ways we can integrate this new information into effective treatment.

## Definition and epidemiology of viral sepsis

### Definition of viral sepsis

Sepsis is a complex syndrome of physiological and pathological abnormalities resulting from infection (1). The pathophysiology of sepsis is not fully understood, making it difficult to give an unambiguous and comprehensive definition of sepsis. The Third International Consensus Definitions Task Force (1) advocated a new definition of sepsis and septic shock in 2015. Sepsis should be defined as “life-threatening organ dysfunction caused by a dysregulated host response to infection,” whereas septic shock is defined as “a subset of sepsis in which particularly profound circulatory, cellular, and metabolic abnormalities are associated with a greater risk of mortality than with sepsis alone.” This version of the definition is designated as “Sepsis-3,” while the previous versions are “Sepsis-1” and “Sepsis-2,” proposed in 1991 (2) and 2001 (3), respectively.

The main differences between Sepsis-3 and previous versions are that Sepsis-3 eliminated the terms sepsis syndrome and severe sepsis, and introduced a new definition of sepsis, which is more comparable to the older definition of severe sepsis. In addition, the systemic inflammatory response syndrome (SIRS) criteria, which were the essential elements of Sepsis-1 and Sepsis-2, are no longer used to define sepsis, but they still play a role in the recognition of infection and warrant early intervention for possible sepsis (1, 4). Sepsis-3 provides a more specific and universal definition for sepsis, which would improve clinical management and facilitate epidemiological surveys. It also has better predictive ability for in-hospital mortality (1).

However, Sepsis-3 is not designed for paediatric populations, which carry a high burden of sepsis (5). The current, widely-used consensus definition of paediatric sepsis, proposed in 2005, was still built on the SIRS criteria (6). It has been shown to lack specificity and perform poorly in identifying children at high risk of mortality from infection (7–9). In addition, its feasibility and applicability when applying to a non-intensive care setting or low- and middle-income countries remain questionable (9). Some evidence has suggested it may be useful to apply the Sepsis-3 definition to paediatric populations (8, 10–12). However, many children with severe viral respiratory tract infections (e.g., bronchiolitis) fulfil the criteria for viral sepsis, but generally clinicians would not regard them as “septic,” highlighting the difficulty in providing a robust definition of viral sepsis (13). Therefore, there is an urgent need to convene a consensus task force and design a paediatric definition (9, 14).

Although bacterial or fungal infections are commonly attributed as the cause of sepsis, sepsis is infrequently attributed to viral infections. In some cases, viral sepsis is regarded as virus-induced direct tissue or cell damage (e.g., influenza virus-induced pulmonary epithelial damage) instead of systemic dysregulation caused by virus. However, the above-mentioned consensus definitions of sepsis, either for adult (1) or paediatric (6) populations are not pathogen-specific, so the same definitions should also apply to viral infection. Therefore, in this review article, viral sepsis is defined as life-threatening organ dysfunction due to a dysregulated host response to viral infection in both adult and paediatric populations. Viral infection can be diagnosed by associated clinical presentations plus positive results of culture, antigen detection, molecular detection (e.g., polymerase chain reaction, PCR), serology, histopathology or immunohistochemistry (15). Viral sepsis should always be considered in septic patients lacking evidence of bacterial, parasitic or fungal infection, and laboratory tests for viruses should be arranged accordingly. In the following sections, we will review the current evidence available about the epidemiology, aetiology, immune pathogenesis, and potential treatments of viral sepsis.

### Burden of viral sepsis

Most of the available large-scale epidemiological studies on sepsis were based on the Sepsis-2 criteria. Severe sepsis defined by Sepsis-2 (3) is similar to the definition of sepsis according to the Sepsis-3 criteria (1). Therefore, where available, we will use the data on severe sepsis from studies based on Sepsis-2 to represent the epidemiological data on sepsis, defined by Sepsis-3.

In general, the incidence and severity of sepsis are climbing over time, whereas sepsis-associated mortality is declining (16–19). A recent systematic review and meta-analysis (17) reported that the global incidence and mortality of hospital-treated sepsis in adult populations were 270 per 100,000 person-years and 26%, respectively, in the last decade. Extrapolating these figures translated to global estimates of 19.4 million sepsis cases and 5.3 million deaths annually. Another global study (5) found that the incidence and mortality of sepsis in paediatric populations (between 4 weeks and 20 years of age) were 22 per 100,000 person-years and 9–20%, respectively; the incidence and mortality of neonatal sepsis (Sepsis-2 definition) were 2,202 per 100,000 live births and 11–19%, respectively. Extrapolation of the data resulted in global estimates of 3 million sepsis episodes in neonates and 1.2 million episodes in paediatric populations annually. Infection-related mortality outside the neonatal period has been falling, but these sepsis episode rates emphasise the importance of focus on tackling sepsis in the first 4 weeks of life.

Both studies (5, 17) indicated several limitations about the epidemiological surveys on sepsis. Firstly, there was no population-level data available from low-income countries, which represent 87% of the world's population (17) and bear a huge burden of infectious diseases and sepsis (20). Therefore, these figures were likely to be underestimates. Secondly, the lack of a universal and specific sepsis definition and severity criteria leads to substantial heterogeneity in case definitions among studies. Lastly, studies may use different denominators to calculate the incidence and mortality (e.g., person-years, specified populations, live births). These hurdles make meta-analysis more challenging and susceptible to bias.

On top of these limitations, most epidemiological studies on sepsis either excluded cases of viral origin or did not specify the proportion of viral sepsis. Organisms that contribute to sepsis can be identified in 59–69% of septic patients (i.e., documented sepsis), with bacteria usually accounting for more than 70% of the documented sepsis cases (21–23). Viruses only contribute ~1% of the documented sepsis cases in some studies (22, 23). However, this figure likely understates the prevalence of viral sepsis for several reasons. A recent Southeast Asian prospective study (24), using a predefined set of laboratory tests (including PCR tests for multiple viruses), demonstrated that viruses accounted for 76 and 33% of the documented sepsis cases (Sepsis-2 definition) in paediatric (excluding neonates) and adult populations, respectively. The most common viruses identified were dengue viruses (27%), followed by rhinovirus (23%), influenza viruses (14%), and respiratory syncytial virus (12%). Although the study was only conducted in tropical middle-income countries and not using the Sepsis-3 definition, it still provides direct evidence that viral sepsis may be underdiagnosed if diagnostic tests for viruses are not performed.

However, the identified viruses could be the single causative agent of sepsis (e.g., dengue), a contributor to secondary bacterial sepsis (e.g., influenza and staphylococcal sepsis) (25), coinfection of unknown significance (e.g., rhinovirus), prolonged or persistent shedding of a previous infection (e.g., adenovirus) (26), an “innocent” latent infection (e.g., Epstein-Barr virus) or a false positive result. This also needs to be taken in the clinical context. For example, a profoundly immunosuppressed child with respiratory symptoms and a high rhinovirus viral load in blood would suggest rhinovirus as the cause of the sepsis. However, a previously well child with purulent meningitis and rhinovirus in a nasal swab would not. This uncertainty is another major obstacle to studying the epidemiology of viral sepsis, particularly in cases where bacterial infection is also documented or the identified virus does not usually cause fulminant disease. What role the identified virus plays in a septic patient is still an area of debate.

Similarly, another prospective study demonstrated that a third of adult patients requiring intensive care for severe pneumonia had viral infections, detected by a predefined array of diagnostic tests (including PCR tests for multiple viruses) (27). Pneumonia is the most common clinical syndrome in patients with sepsis (21, 22, 28). Moreover, another prospective study found that patients with culture-negative sepsis had significantly lower levels of procalcitonin than those with documented sepsis (21). It has been shown that elevated procalcitonin levels are more likely to be seen in bacterial infections than viral infections and may be used to differentiate infections caused by bacteria and viruses (29, 30). Therefore, these studies suggest that viruses may cause more sepsis cases. The exact incidence of viral sepsis remains to be elucidated.

The World Health Organization and the World Health Assembly have listed sepsis as one of the global health priorities for the following years (31). They also recognized the importance of understanding the epidemiological burden of sepsis. In order to obtain a comprehensive picture of the burden of sepsis, there is an urgent need to understand the epidemiology of viral sepsis.

## Aetiology of viral sepsis

Almost any virus can cause viral sepsis in susceptible populations (24). Herpes simplex virus (HSV) and enteroviruses are the most common viral causes of neonatal sepsis (32), while enteroviruses and human parechoviruses (HPeVs) are the most common causes of viral sepsis in young children (33). In addition, influenza viruses are not only a major cause of severe infections and deaths among children younger than 5 years of age, older adults, pregnant women and immunosuppressed individuals (34), but can also lead to substantial morbidity and mortality in older children and adults in other age groups (35). Furthermore, dengue viruses are a leading cause of sepsis in some tropical countries (24). We will review the characteristics and epidemiology of these prominent causes of viral sepsis in the following section.

### Herpes simplex viruses

HSV is one of the leading causes of neonatal sepsis (32). In neonates, HSV can cause three types of disease: skin, eye and mouth disease, encephalitis, and disseminated disease (36). Disseminated HSV disease is the most severe form of HSV infection, with a case fatality rate of as high as 29% (29). Patients with lethargy, severe hepatic dysfunction or delayed treatment have higher mortality (29). HSV can also cause fulminant hepatitis in non-neonatal populations, typically without obvious cholestasis (37). Both disseminated HSV disease and fulminant HSV hepatitis have a clinical presentation of viral sepsis, involving hepatic dysfunction, respiratory failure, disseminated intravascular coagulopathy, and haemodynamic instability (36, 38). In the absence of skin lesions, disseminated HSV disease and HSV hepatitis are difficult to differentiate clinically from sepsis caused by other pathogens (36, 38).

Studies have reported various incidence rates of neonatal HSV infection, ranging from 8 to 60 per 100,000 live births, with disseminated HSV disease accounting for 25% of cases (39). Some viral factors are associated with viral sepsis. Firstly, a large inoculum of HSV may increase the risk of viral sepsis (38). Secondly, it has been shown that maternal genital HSV type 1 (HSV-1) infection has a higher probability of transmission to neonates during labour than HSV type 2 (HSV-2) infection (40). However, HSV-2 accounts for a higher proportion of neonatal central nervous system (CNS) and disseminated HSV diseases although HSV-1 causes about 60% of cases of neonatal HSV infection (41). This also explains why HSV-2 is associated with higher morbidity and mortality (39). Furthermore, neonates born to mothers with newly-acquired genital HSV infection near term are at greater risk for neonatal HSV infection than those born to mothers with reactivated genital HSV infection (42).

### Human enteroviruses

Enterovirus is a genus of viruses of the family *Picornaviridae*, which have been shown to cause sepsis in immunodeficient and paediatric populations (43). Enteroviruses are the causative agents of a broad range of clinical conditions including aseptic meningitis and myocarditis, although many cases are asymptomatic or benign (44). There are about 10–15 million symptomatic enteroviral infections in the United States per year (43) with a disproportionately high number of infections occurring in neonates (11.4–11.6% compared with the average yearly birth cohort percentage of 1.5%) (45, 46). The enteroviruses that have the highest association with sepsis are coxsackievirus and echovirus (47); these viruses primarily cause sepsis in neonates (47). In contrast, enterovirus A71 can lead to viral sepsis in children beyond the neonatal period (48, 49), predominantly in children younger than 2 years of age (50).

Risk factors for neonatal enteroviral infection include exposure to maternal secretions or blood during delivery, maternal infection just before or at delivery, and a lack of previous maternal infection by the infecting serotype, resulting in low maternal antibody levels against that serotype of virus. The majority of severe neonatal enteroviral infections occur between days 3 and 5 of life, suggesting that the acquisition is generally in the perinatal period and is preceded by maternal infection (51).

### Human parechoviruses

HPeVs are also frequently associated with sepsis in paediatric and immunodeficient populations. HPeVs have previously been defined as a subset of the genus *Enterovirus* but were eventually re-classified as their own genus of viruses after sequencing revealed them to be unrelated to enterovirus. Antibodies against HPeVs are present in the cerebrospinal fluid (CSF) of up to 99% of the population (52), with HPeV type 3 (HPeV3) (the most common cause of HPeV sepsis) being present in 10% of the population studied in the Netherlands and 13% in Finland (52). HPeVs are the second most common cause of viral sepsis in young children after enteroviral infections (33). HPeV infections are often asymptomatic or present with very mild symptoms, although severe infections can have symptoms ranging from sepsis and sepsis-like illnesses to viral meningitis and encephalitis (33). White matter abnormalities on magnetic resonance imaging have been found in cases of HPeV encephalitis, which may play a role in the development of sequelae (53–55). The development of white matter abnormalities and sequelae seems to show little association with short term outcomes (54). Clinical presentations of HPeV infections are similar to those of enteroviral infections, are clinically indistinguishable and require serology or PCR to discriminate between them. HPeV3 is the HPeV most commonly associated with severe disease, with other HPeVs known to cause severe disease only rarely (56, 57).

Licensed specific antiviral therapy is not available for HPeV or enterovirus infections, despite their relatively high incidence in neonatal encephalitis and systemic infections (58). This presents a promising target for future research, and a tangible way to reduce the incidence of neonatal viral sepsis and associated infant mortality globally.

The addition of a PCR test for HPeVs to the re-analysis of 761 banked CSF samples from children presenting with sepsis found a 31% increase in detection of a viral cause of sepsis in these cases (33), with HPeV being found in 0.4–8.2% of neonates presenting with sepsis, depending on the year, with an overall detection rate of 4.6%. It is thus likely that viral sepsis caused by HPeVs is frequently underdiagnosed.

### Influenza viruses

Influenza A and B viruses cause seasonal epidemics and out-of-season outbreaks worldwide (34). Influenza sepsis can present as severe pneumonia, acute respiratory distress syndrome (ARDS), myocarditis or encephalopathy. The estimated annual incidence proportions of influenza virus infection are 5–10% in adults and 20–30% in children (34). A modelling study demonstrated that seasonal influenza epidemics account for an estimated 290,000–650,000 respiratory deaths annually, with 10,000–110,000 occurring among children younger than 5 years of age (59). The true mortality attributable to influenza viruses must be higher because the figures do not include deaths from other causes, such as circulatory deaths (59), which also make up a large proportion of influenza virus-associated deaths (60). Approximately 60% of mortality from seasonal influenza occurs in people older than 65 years of age (59), while 80% of the non-survivors in the 2009 influenza H1N1 pandemic were people younger than 65 years of age (61). The age shift may be explained by some level of immunity to H1N1 strains in people born before 1957, when H1N1 strains widely circulated and had not been replaced by the H2N2 pandemic strain (35). In addition, a study (62) found high titres of low-avidity, non-protective immunoglobulin G against the viral H1 antigen in severely ill middle-aged adult patients. Pulmonary immune complex deposition and complement activation were also observed. This provides evidence that immune complex-mediated disease may be part of the pathogenesis of severe pneumonia in middle-aged patients, which also contributes to the age shift.

The pathogenesis of influenza virus infection depends on viral virulence and host responses (63). Host responses will be discussed in the next section. The crucial site for influenza virus infection that leads to severe pneumonia is the alveolar epithelium (64). Haemagglutinins (HAs) of different strains of influenza viruses have varied tropism for the airway epithelium. For example, seasonal influenza viruses bind preferentially to the epithelium in the upper airway and bronchi and, to a lesser extent, to the alveolar epithelium (65, 66). By contrast, HA of the 2009 pandemic influenza A (H1N1) virus attaches to both type 1 and type 2 pneumocytes, while HA of the avian influenza H5N1 virus primarily binds to type 2 pneumocytes (64). The second determinant of viral virulence is the viral polymerase complex, which is associated with different levels of viral replication and cytokine production in the infected epithelial cells (65). Therefore, the differing tropism, along with the varied degrees of viral replication and cytokine production, induces various extents of cell death and in part explains the differences in pathogenicity between different strains (63, 67).

### Dengue viruses

Dengue is a common viral infection in tropical countries and has the capacity to cause viral sepsis. Dengue viruses are currently considered the most important and widespread virus spread by mosquitos (68). Over 50% of the world's population live in areas where dengue infection occurs (68). Estimates of yearly dengue infections range from 50 million up to 400 million (68). Dengue viruses are members of the family *Flaviviridae*, with four distinct serotypes; serotypes 1, 2, 3, and 4 (69). Infection with any of these serotypes gives full protection against that serotype; however, after infection with one serotype, infection with any of the others can result in an enhanced and more severe form of the disease (70). All serotypes of dengue viruses have been implicated in severe dengue (68).

One study in Thailand found that ~14% of patients diagnosed with sepsis (Sepsis-2 definition) tested positive for dengue viruses upon re-analysis of banked serum samples by PCR (71). Of the patients who were diagnosed with dengue by PCR from banked serum samples five had died, of which four had been diagnosed with sepsis but not dengue infection. It was suggested that it may prove beneficial to increase testing for dengue viruses in patients presenting with sepsis to ensure the patient receives appropriate treatment (71).

Table 1 summarises the clinical syndromes, epidemiology and risk factors of sepsis caused by these viruses and adenovirus. While being the most commonly detected viruses causing sepsis, they are far from the only ones. Other viruses, such as chikungunya virus (84), hantavirus (85), coronaviruses (86), Ebola virus (87), and Lassa virus (88), among many others, are also major contributors to viral sepsis across the globe. Due to the limited data on many of these viruses, the immune responses against them and their pathogenesis are poorly understood. In addition, these viruses do not occur at a high enough incidence in populations in high-income countries to gain significant research funding. This may change in future as more effective treatments are discovered for more frequently occurring infections and less common diseases become more attractive to research.

**Table 1:** Summary of the clinical syndromes, epidemiology and risk factors of sepsis caused by different viruses.

	**Clinical syndromes**	**Epidemiology**	**Risk factors for sepsis**	**References**
HSV	Disseminated disease Hepatitis	Neonatal disseminated disease Incidence: 2–15 per 100,000 live births Mortality: up to 29%	Newly-acquired maternal genital infection near term HSV-2 infection (compared with HSV-1 infection)	(29, 38, 39)
Enterovirus	Sepsis-like illness Myocarditis Encephalomyelitis Pulmonary oedema or haemorrhage	Incidence: 37% of young infants (<90 days of age) with sepsis^a^ and without signs of localised infection Mortality of neonatal enteroviral sepsis: up to 42%	Lack of maternal antibodies Maternal infection just before or at delivery Neonatal infection with echovirus 6, 9, 11, 19 or coxsackievirus B2–B5 Enterovirus A71 infection in young children	(29, 48, 72, 73)
HPeV	Sepsis-like illness Meningoencephalitis	Incidence: 15% of young infants (<90 days of age) with sepsis^a^ and without signs of localised infection	HPeV3 infection (compared with infection with other types of HPeV)	(33, 73)
Influenza virus	ARDS Myocarditis Encephalopathy	Incidence: 1 million cases of severe respiratory infections in children <5 years of age worldwide annually Mortality: 290,000–650,000 respiratory deaths worldwide annually (all age groups)	People of extreme age (<5 or >65 years) Immunosuppression Pregnancy Influenza A (H3N2) virus infection (compared with influenza A (H1N1) or B virus infections)	(34, 59, 74, 75)
Dengue virus	Severe dengue^b^ Dengue shock^b^	Incidence: 58–96 million symptomatic dengue infections with 250,000–500,000 progressing to severe disease worldwide annually; 8% of sepsis cases^c^ in Southeast Asian Mortality: 9,000–24,000 deaths worldwide annually	Previous dengue infection (with a different serotype)	(20, 24, 70, 76–79)
Adenovirus	Disseminated disease Meningoencephalitis Severe pneumonia	Disseminated disease in children Incidence: 2.5% of adenovirus infection Mortality: 55%	Immunosuppression (particularly allogeneic HSCT) Young children Infection with adenovirus serotypes 3 and 7	(80–83)

a *Sepsis was defined according to age-specific criteria, Rochester criteria and Yale observation scale*.

b *2009 WHO revised dengue case classification*.

c *Sepsis was defined by the Sepsis-2 definition*.

*HSV, herpes simplex virus; HPeV, human parechovirus; ARDS, acute respiratory distress syndrome; HSCT, haematopoietic stem cell transplants*.

## Susceptible populations

Neonates and young children (89), pregnant women (90), older adults (89), and immunosuppressed individuals (91) are especially susceptible to severe infections and sepsis. Here, we will review current evidence regarding the immunological characteristics of these susceptible populations that predispose them to severe infections, especially viral sepsis.

### Neonates and young children

The immature and naïve immune system of neonates predisposes them to infection with intracellular pathogens and sepsis (92, 93). One of the most remarkable features of the neonatal innate immune system is the bias in favour of type 2 helper T (T_H_2)-cell responses, which results in reduced secretion of pro-inflammatory cytokines, such as interleukin (IL)-12, tumour necrosis factor (TNF), interferon (IFN)-γ, and IL-1β, which together with immature innate immunity allows pathogens to replicate and spread more easily (93, 94). In contrast, neonatal monocytes and antigen-presenting cells display preserved or even enhanced Toll-like receptor (TLR)-mediated production of some cytokines (e.g., IL-6, IL-10, and IL-23) (93). In addition, studies have shown that neonates can experience highly exaggerated inflammatory responses through some pathways, such as the TLR2 pathway, in response to specific antigens (95, 96). These exaggerated responses may play a role in the development of sepsis in response to viral infections.

There are also other features of the neonatal immune system that increase susceptibility to severe viral infection. Firstly, neonatal monocytes have decreased expression of the major histocompatibility complex (MHC) class II, which leads to impaired antigen presentation (93). Secondly, neonatal dendritic cells have a reduced production of TNF and type I IFNs, impaired upregulation of CD80 and CD86 co-stimulatory molecules, and reduced stimulation of T cell proliferation, all of which can contribute to a decreased ability to clear viruses (93). In addition, neonates have low levels of complement components (93); complement is responsible for antibody-independent opsonization and lysis of pathogens and plays a role in the activation and enhancement of the adaptive immunity against infections (97). Furthermore, there are both quantitative and qualitative defects in neonatal neutrophils (93). For example, lower levels of neutrophils in stress situations, such as sepsis, are seen in neonates. The qualitative deficiencies in neonatal neutrophils include impairment of adhesion, migration, chemotaxis and amplification. These defects lead to a reduced ability to clear viruses and other pathogens. Lastly, the naïve, immature adaptive immunity of neonates, together with the lack of pre-existing immunological memory, increases their susceptibility to various pathogens and severity of infection (94).

It is worth noting that susceptibility to sepsis persists beyond the neonatal period (i.e., 4 weeks of age). Young children also bear a substantial burden of sepsis, with the peak incidence of 516 per 100,000 population in the infant group (Sepsis-2 definition) (5). Likewise, hospitalisation rates for viral infections, such as influenza (98) and respiratory syncytial virus infection (99), are highest in children younger than 2 years of age. In addition, children with underlying diseases (e.g., bronchopulmonary dysplasia, congenital heart diseases, neurological disorders) are at greater risk of developing severe viral infections and sepsis (98, 100, 101).

### Pregnant women

Pregnant women are another population at greater risk of viral sepsis than the general population. There have been several reports on maternal sepsis caused by influenza, herpes simplex, varicella-zoster, and chikungunya viruses, among others (102–106). Recognition of the problem of influenza related mortality in late pregnancy has led a number of countries to introduce routine influenza vaccination in pregnancy. According to the Global Burden of Disease Study, there were an estimated 17,900 deaths from maternal sepsis and other infections globally in 2015, accounting for 6.5% of the total deaths from maternal disorders (20). The incidence of maternal sepsis is around 41–49 per 100,000 pregnancies with a mortality rate of 1.8–4.5% in the United Kingdom (105) and the United States (107). An increasing trend in the incidence and mortality of maternal sepsis has been seen in the recent decades (108). However, these studies did not report the proportion of women with viral sepsis.

The maternal immune system is complicated and delicately modulated. It is tolerant to paternal antigens and the “allogeneic” foetus, while efficient at identifying and defending against invading pathogens to protect the mother and the foetus (109). The immunological characteristics during pregnancy depend on the stage of gestation and the area of focus. For example, a pro-inflammatory [type 1 helper T (T_H_1)-biased] status with high levels of IL-6, IL-8, and TNF-α is seen in pregnant women during the first trimester of pregnancy, which is critical for embryo implantation, placentation and initial foetal growth. Following this, pregnant women develop a more anti-inflammatory (T_H_2-biased) status with increased levels of prostaglandin E_2_, IL-4, and IL-10 while the foetus grows rapidly. Before labour, the immune system shifts back to a pro-inflammatory (T_H_1-biased) status, which helps parturition (110). Additionally, the epithelial cells of the reproductive tract are down-regulated with low levels of IL-1β, IL-8, and IL-6 in cervical fluid (109). Pregnant women also encounter reduced levels of immunoglobulin G (111) and a decreased number of helper T lymphocytes (111) throughout pregnancy.

The maternal immune system is not yet understood completely. However, we do know that it is constantly changing, and not just universally suppressed. The unique immune profiles result in different responses to pathogens, which may make pregnant women more susceptible to some pathogens depending on the stage of pregnancy (110). In addition, the immune response originating from the placenta also influences the maternal immune response to microorganisms. For example, an insult such as a subclinical viral infection of the placenta can affect the maternal immune system and increase the maternal susceptibility to various pathogens, including viruses (112).

### Older adults

Older adults (>60 years old) are a population at a significantly greater risk of sepsis from all causes than the general population (113). Older adults were found to have an incidence of sepsis of 26.2 cases per 1,000, which is considerably higher than the 3.0 cases per 1,000 observed in the general population (114). There are many reasons for this increased susceptibility, including a higher likelihood of co-morbidities, prolonged hospitalisation times, generally weaker immune responses, and immunosenescence (115). Viral sepsis in older adults presents one of the most serious upcoming global health problems, as the global population of older adults is set to overtake the “young” population by 2050. It is therefore important to understand the unique conditions within older adults to better facilitate the development of suitable treatments. Many of the factors involved in the increased susceptibility of older adult populations to sepsis also increase the susceptibility to viral infection, and thus viral sepsis.

Comorbidities for sepsis and severe viral infection such as diabetes mellitus (116), renal failure (117, 118) chronic obstructive pulmonary disease (117, 119), heart conditions (120), and obesity (120, 121) are much more prevalent in older adult populations. There are many other comorbidities in older adults that can increase the risk of viral sepsis. These often are associated with immunosuppression [e.g., renal failure (122)].

Immunosenescence is the gradual deterioration of the immune system brought on by advancing age, which increases susceptibility to both viral infections, and the development of viral sepsis. It is characterised by a decrease in the function of phagocytes (123–125), antigen presentation (124) and lymphocytes (126, 127), as well as decreased cellular replication (128, 129) and ability to respond to cytokine stimulus (130). Older adults also experience persistent T cell exhaustion in part due to constant low-level inflammation, thought to be caused by accumulation of self-debris brought on by a decrease in the ability to clear them (131). This process, often called “inflammaging” is characterised by elevated baseline levels of the cytokines IL-6, IL-1, and TNF-α (130, 132). Another factor contributing to T cell exhaustion in older adults is the prolonged length of inflammatory states after infections (133) which can result in decreased T cell replication and inhibition of co-stimulation by antigen presenting cells. This results in a decreased ability to effectively respond to infection, allowing viral infections to easily evade eradication by the immune system and develop into serious systemic infections.

### Immunosuppressed individuals

The number of immunosuppressed hosts has grown considerably over the last decades due to the widespread use of cytoablative chemotherapy, monoclonal antibodies and immunomodulatory agents for neoplastic and autoimmune diseases, the epidemic of human immunodeficiency virus (HIV) and increasing numbers of haematopoietic stem cell transplants (HSCT) and solid organ transplants (SOT). The clinical picture of sepsis in immunosuppressed hosts is usually diminished or non-specific, making it difficult to diagnose or distinguish from other non-infectious causes, such as transplant rejection (91, 134). Thus, infection and sepsis continue to be the major cause of morbidity and mortality in immunosuppressed hosts (91, 135). A multicentre, prospective study (135) in the United States showed that 42% of HSCT recipients had viral infections at some point post-transplant (median follow-up, 413 days), and infection accounted for 21% of deaths. However, the authors did not specify the mortality rate caused by viral infections.

Individuals with neutropenia or taking corticosteroids mainly have impaired innate immunity, while transplant recipients primarily have defects in adaptive immunity (91, 136). Similar to immunocompetent hosts, many viruses are able to cause sepsis in immunosuppressed hosts, but some are of particular concern. For example, HSCT and SOT recipients are at high risk of infection with cytomegalovirus (CMV), other herpesviruses and respiratory viruses (e.g., adenovirus, influenza virus, parainfluenza virus and respiratory syncytial virus) (136). In transplant recipients, the timing of viral infections varies according to the types of transplant, antimicrobial prophylaxis received, and other host and donor factors, but they are most likely to occur 1 month after transplantation when defects in cell-mediated immunity dominate or graft-versus-host disease occurs (134, 137). By contrast, the risk of infection with CMV, other herpesviruses, and respiratory viruses may be lower in neutropenic hosts, patients with HIV infection and individuals taking corticosteroids than in transplant recipients (136).

Primary immunodeficiency is another special category of diseases comprising at least 200 genetic disorders of variable severity (138) and affecting more than six million people worldwide (139). This population is at substantial risk of disseminated viral infection and sepsis. Young children with inborn errors in signalling pathways upstream of the production of type I IFNs are at higher risk of developing life-threatening viral infection (140). For example, signal transducer and activator of transcription 1 (STAT1) or nuclear factor (NF)-κB essential modulator (NEMO) deficiency leads to lethal HSV disease and various other severe viral infections (140–142). Deficiency in interferon regulatory factor 7 also leads to more severe viral infections due to decreased downstream IFN signalling (143). Other disorders that have been demonstrated to have an effect in viral infections are the IFITM3 SNP rs12252, which affects CD8+ T cell numbers (144), and variations in receptor components such as the IFIH1 receptor, which decreases downstream signalling to IFNs (145). In addition, patients with severe combined immunodeficiency have major defects in B and T lymphocyte development, facing a substantial risk of infection caused by a wide variety of pathogens (e.g., fulminant adenovirus and HSV infections) (146, 147).

## The immunology of viral sepsis

### Normal immune responses to viral infection

Pattern recognition receptors (PRRs) are responsible for the initial detection of viruses (148). They can recognize pathogen-associated molecular patterns (PAMPs) (e.g., viral RNA and DNA) and damage-associated molecular patterns (DAMPs) (e.g., host DNA and proteins) (148). There are several families of PRRs, such as TLRs, cytosolic RNA sensors [e.g., retinoic acid–inducible gene (RIG)-I and melanoma differentiation–associated gene 5 (MDA5)] and cytosolic DNA sensors (e.g., absent in melanoma 2, IFN-γ-inducible protein 16, and cyclic GMP-AMP synthase) (149). When encountering pathogens, PRRs play a critical role in the activation of innate immune responses and the recruitment of leucocytes (148, 149).

**Figure 1:**
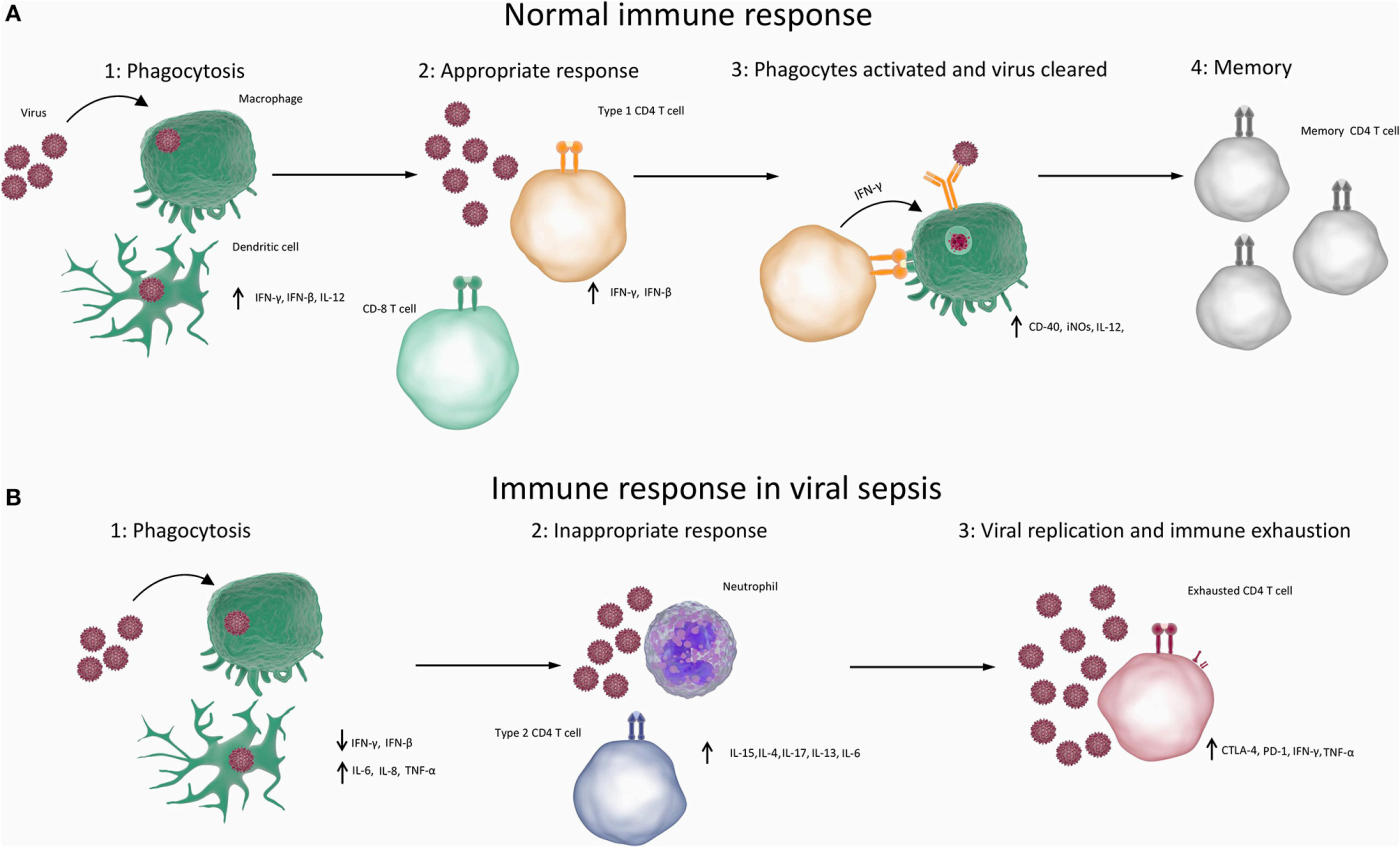
Immune responses in viral sepsis. **(A)** Normal competent response to viral infection resulting in clearance of infection **(1)** When the immune system is exposed to a virus, the virus infects or is phagocytosed by macrophages, dendritic cells or other phagocytes. Phagocytes break down, process and present antigens from the virus and produce type 1 cytokines. **(2)** Type 1 cytokines cause T cells to differentiate into T_H_1 cells and CD8 T cells. **(3)** T_H_1 cells and CD8 T cells cause apoptosis of infected cells and activate processes such as the production of reactive oxygen species in phagocytes, which destroy the viruses. Antibody production is elevated, resulting in opsonisation, greater phagocytosis and destruction of viruses. **(4)** Virus is cleared and memory T cells are produced, which can rapidly respond to future infections. **(B)** Aberrant immune response resulting in viral sepsis and failure to clear virus. **(1)** When the immune system is exposed to a virus, the virus infects or is phagocytosed by macrophages, dendritic cells, or other phagocytes. Phagocytes break down, process and present antigens from the virus. Non-type 1 cytokines are produced. **(2)** Non-type 1 cytokines result in inappropriate type 2 or type 17 immune responses, which cause inflammation but cannot clear the virus. **(3)** T cells become exhausted and can no longer competently clear pathogens. (CTLA-4, cytotoxic T-lymphocyte–associated antigen 4; IFN, interferon; IL, interleukin; iNOS, inducible nitric oxide synthase; PD-1, programmed death 1; TNF, tumour necrosis factor).

First of all, the innate responses stimulate the production of pro-inflammatory cytokines and have an immediate antiviral effect on preventing virus spread and replication, which is mainly exerted by type I IFNs (150, 151). Furthermore, PRRs can trigger the development of virus-specific adaptive immunity (e.g., cytotoxic T lymphocytes, antibodies) to clear viruses and virus-infected cells (152). Lastly, PRRs can induce the secretion of anti-inflammatory cytokines such as IL-10 and IL-13, which help to resolve the pro-inflammatory state and promote tissue repair (149, 153, 154). The normal immune responses to viral infection are summarised in Figure 1A.

### Hyper-inflammatory phase of viral sepsis

Although pro-inflammatory cytokines are essential to mediate innate immunity, they (particularly IL-6) can cause host damage (149). The release of DAMPs from the damaged tissues and cells can further stimulate PRR signalling and lead to a chain reaction culminating in viral sepsis if the infection is not cleared (155).

#### Herpes simplex viruses

TLR2 and TLR9 comprise the major PRR signalling pathways activated in response to HSV infection (96, 156). Many of the clinical features of viral sepsis caused by HSV can be attributed to exuberant responses induced through TLR signalling (95). It has been demonstrated that levels of IL-6 are negatively associated with the survival of HSV encephalitis (157). Neonatal cord-blood cells mount higher levels of pro-inflammatory cytokines (IL-6 and IL-8) when challenged with HSV than adult blood cells (95). Therefore, HSV causes a higher ratio of IL-6 to TNF in neonates, which contributes to severe inflammation and the development of sepsis (93).

Dysregulated secretion of pro-inflammatory cytokines in response to HSV infection also induces the production of high mobility group box 1 protein (HMGB1) from injured cells (158). HMGB1 is a nuclear protein and regulates DNA transcription. HMGB1 can mount a pro-inflammatory cytokine response to pathological levels and lead to the release of cytochrome c (158). It has been demonstrated that the peak of HMGB-1 comes before the peak of cytochrome c in a clinical case of neonatal disseminated HSV disease (158). Cytochrome c subsequently activates caspase-3 and caspase-7, resulting in extensive apoptosis (149). Apoptosis is responsible for the development of multiorgan dysfunction in septic patients (159).

#### Human enteroviruses

Human enterovirus infections are characterised by a type I IFN response, induced by PRRs that respond to RNA viruses. Enterovirus detection has been found to primarily involve the TLR and RIG-I-like receptor (RLR) signalling pathways. In particular TLRs 3 (160), 7 (161), 8 (162), and 9 (163) have been implicated in the innate response to enteroviruses. The RLR most commonly associated with enterovirus infection is MDA5. MDA5 is involved in detecting intracellular RNA viruses (164) and has been found to play a small role in the development of the innate immune response to enterovirus infection (165). Although other PRR signalling cascades are likely to have a role in sensing enteroviruses, this role has not yet been characterised. Human enteroviruses employ a diverse range of strategies to evade the immune response and replicate. These strategies are critical to the ability to cause viral sepsis, as they allow the virus to replicate sufficiently to cause significant inflammation. Some broad strategies of immune evasion used by enteroviruses are interference with innate immune signalling by either interfering with or avoiding initial PRR recognition [poliovirus can subvert MDA5 signalling and induce apoptosis of innate immune effector cells (166)] or interfering with the downstream cytokine signalling pathways. There is evidence that enterovirus A71 can interfere with the IFN signalling pathway at several points (167). These immune evasion mechanisms have been shown to have a direct impact on survival in mouse models, with blockage of type I IFN pathways in enterovirus A71 infections resulting in increased mortality and viral load (168). Severe infections with coxsackievirus have been associated with host expression of the decay-accelerating factor (DAF) and coxsackievirus and adenovirus receptor (CAR), which have been shown in mouse models to facilitate coxsackievirus infection of neural stem cells (169, 170). DAF and CAR are cell surface receptor proteins involved in the complement pathway (171) and cell adhesion (172), respectively. The role of DAF in disease is not known (170, 173). CAR functions as a receptor which can be used by coxsackieviruses to enter the cell (170). The immune response to enteroviral infections is varied and strain specific; however, a general strong pro-inflammatory response involving IL-1α, IL-1β and TNF-α, alongside an increase in the expression of innate immune receptors for double stranded RNA is observed in most cases (174). These responses alongside virally induced lytic cell death can result in extensive necrosis, further compounding the inflammatory response and potentially leading to conditions of sepsis (174). Interestingly, some strains of enterovirus, such as echovirus-9, seem to cause a particularly high degree of necrosis in pancreatic β islet cells, resulting in a strong correlation between enteroviral infections and diabetes (174).

#### Human parechoviruses

HPeV3 infection has been shown to initiate distinct innate immune responses in CNS infections to those of enteroviral infections despite their similar clinical presentations (175). Enteroviral responses are generally stronger; levels of almost all cytokines are higher in enteroviral responses than in HPeV infections. HPeV infections also do not demonstrate the strong type I IFN responses generally induced by RNA viruses. In addition, HPeV infections demonstrate a lower level of IL-6 expression than both enterovirus infections and controls, suggesting some method of viral immune evasion (175), resulting in these muted immune responses. The markedly lower innate immune cytokine responses induced by HPeV3 infection are interesting, as HPeV3 has a similar clinical presentation to that of many enteroviral infections, raising questions about disease pathogenesis and viral classification. It has also been shown that HPeV is detected by TLRs 7 and 8 (176) which activate the type I IFN pathway (177). There is evidence that HPeVs (in particular HPeV type 1) employ methods to dampen type I IFN signalling (178) in a cell type dependent manner. Treatment of these cells with type I IFN, however, was not found to inhibit viral infection of these cells. It could be interesting to explore the relevance of this evasion mechanism in the context of a systemic infection. The immune response to HPeV infection is not as well-characterised as that of enteroviral infection; however, certain constants, such as the suppression of type I IFNs as an immune evasion mechanism, indicate that type I IFN pathways are important in the resolution of these infections. There are few data analysing cytokine populations of these infections in the context of sepsis, so while it is possible to characterise the immune responses involved in these infections, it is not known if these hold true in viral sepsis.

Several reasons have been proposed for why HPeV3 is more likely to cause severe infections than other HPeVs. It has been observed that HPeV3 lacks a sequence motif present in other HPeVs that is thought to play a role in viral use of integrins as host cell receptors, suggesting that the virus may be exploiting a different receptor to enter cells. This may allow it to more easily access host cells and thus replicate to a higher degree than other HPeVs (179). It has also been noted that HPeV3 is not efficiently neutralised by antibodies naturally generated against it, while HPeV type 1 was efficiently neutralised, which may explain why HPeV3 infections tend to be more severe. Maternal antibodies would be insufficient to neutralise the virus in a neonate, resulting in more severe, prolonged infections (180).

#### Influenza viruses

Recognition of influenza virus is primarily through TLR3 (181), TLR4 (182), TLR7 (183), and RIG-I (184), which signal through NF-κB to induce subsequent immune responses (185). The polymorphisms of these receptors in different individuals in part determine their susceptibility to, and the severity of, influenza virus infection (186). The disruption of the alveolar epithelial-endothelial barrier, leading to pulmonary oedema and further respiratory insufficiency, is essential to the development of severe pneumonia and ARDS caused by influenza virus (64). Influenza virus first infects alveolar epithelial cells rather than alveolar endothelial cells, which are usually the primary target for bacteria-induced ARDS (64, 187). Influenza virus can cause apoptosis of the epithelial cells by the upregulation of the *Fas* gene via activating protein kinase R (188). In addition, the infected epithelial cells produce a broad range of pro-inflammatory cytokines, such as TNF-α, IL-1β, IL-6, IL-8, CCL5 (RANTES), and CXCL10 (IP-10) (64). These cytokines can damage the epithelial-endothelial barrier through mechanisms that are not yet fully understood (64). Studies have demonstrated that IL-1β and TNF-α can cause decreased activity of amiloride-sensitive epithelial sodium channels, one of the key ion channels that clear alveolar fluid (189). TNF-α has also been shown to be able to disrupt the tight junctions between epithelial cells (190).

Some of the cytokines that are produced by epithelial cells can cause the recruitment and extravasation of monocytes and neutrophils by direct chemotaxis or upregulating adhesion molecules (e.g., P-selectin, E-selectin) on the endothelial cells (64). Newly-recruited neutrophils and macrophages, derived from monocytes, can produce a wide variety of pro-inflammatory cytokines, reactive oxygen species and nitric oxide (by inducible nitric oxide synthase), all of which further damage the barrier (64). These cytokines can also recruit more neutrophils and monocytes into the alveolar lumen (64). It has also been shown there is a positive correlation between the concentration of neutrophils in bronchoalveolar lavage fluid and the severity of ARDS (191). In addition, macrophages produce IFN-β in a protein kinase R- and NF-κB-dependent fashion and express TNF-related apoptosis-inducing ligand (TRAIL) by the stimulation of IFN-β (192). The interaction between TRAIL and death receptor 5 on epithelial cells is another mechanism for the apoptosis of epithelial cells (64).

#### Dengue viruses

The innate immune response to dengue infection is primarily characterised by an IL-8 and type I IFN response activated by TLR3 after the virus is degraded by endosomal acidification (193). Dengue virus also activates the TLR7, TLR8, RIG-I and MDA5 signalling pathways, which signal through type I IFNs (194–196). There is also evidence that dengue virus may signal through TLRs 2 and 4, and that this may contribute to the pathogenesis and extreme inflammation of severe dengue (197). Type I IFN induction is important for a successful immune response to dengue virus (198). Many of the documented immune evasion mechanisms employed by dengue virus involve the inhibition of type I IFN signalling by some method. Dengue virus has been demonstrated to inhibit IFN production by a RIG-I dependent mechanism (199). Inhibition of IFN production contributes to the ability of dengue virus to replicate and spread, and thus to cause severe systemic infections and viral sepsis. Dengue virus can also cause an antibody enhanced form of disease when a patient is exposed to a different serotype of dengue virus than the one with which they were initially infected (70). Enhanced dengue disease involves the same immune responses as the non-enhanced form of disease, with a key difference being the presence of cross-reactive antibodies, which do not neutralise the pathogen but instead allow the virus to replicate within Fc receptor containing cells, resulting in a more severe infection (200).

### Immune suppressive phase of viral sepsis

Figure 1B summarises the aberrant immune responses in viral sepsis. It has become apparent in recent years that the immunosuppression that results from sepsis may contribute more to mortality than the initial hyper-inflammatory response. A more immunosuppressive genotype in patients with sepsis is correlated with increased mortality (201, 202). It has also recently been reported that the majority of deaths in adults [68%] that occur during sepsis from all causes happen on the third day or later (203). 20.4% of these deaths could be attributed to nosocomial infections. Independent predictors of third day or later death were corticosteroid treatment, no identification of the pathogen (203), and age. These risk factors differ in paediatric populations, with most deaths occurring within 48 h of presentation (204, 205). Most deaths in children occurred due to refractory shock (204). This suggests that in adults most sepsis related deaths are not due to an initial overpowering immune response, rather due to an inability to control infections that result in excessive pathogen proliferation and inflammation, whilst children are less able to survive an initial fulminant infection.

The initial inflammatory immune response that characterises viral sepsis is usually followed by a period of immune suppression. This phenomenon is characterised by decreased function in both innate and adaptive immunity, with common features including increased expression of negative co-stimulatory molecules and decreased expression of positive co-stimulatory molecules, T cell exhaustion, apoptosis of effector cells, increased regulatory T cell expression and higher numbers of myeloid derived suppressor cells (206, 207). This can result in increased infections from secondary pathogens, but also the reactivation of dormant infections, and natural microbiota becoming pathogenic (206). In particular, the reactivation of herpesviruses such as CMV and HSV have been found to occur in 33 and 21% of immunocompetent patients with severe infections requiring hospitalisation, respectively (208, 209). Due to the inability of the immune system to adequately control or eradicate these infections they can often result in severe disease (210), tissue damage, and even death. Mortality in sepsis is often caused by serious secondary infection after the initial inflammation has already passed. Immune suppression is a common feature of persistent and serious viral infections (211).

Some viral infections can directly result in immune suppression. Some strains of enterovirus are known to infect leucocytes (212). In particular some strains, such as coxsackievirus B3 and enterovirus 70, that have been implicated as causes of sepsis have shown this capacity (212). Infection of leucocytes in severe viral infections has been shown to result in an immunosuppressed state, as the death of infected leucocytes can result in a diminished ability to prime CD4 and CD8 cells and a reduced ability to control pathogens by phagocytosis. This greatly increases the susceptibility of the host to secondary infections (210). When compounded with other immune suppressive effects of severe infections, such as lymphocyte exhaustion, the ability to handle new infections can become drastically reduced (211). This phenomenon could explain the higher mortality observed in infections of relatively immunosuppressed patients, as well as the high level of mortality observed in sepsis after the initial inflammatory response has passed.

Serious infections that cause prolonged inflammation very often result in immune exhaustion. It was found in *in vivo* experiments that prolonged infections by a large variety of viruses can result in decreased differentiation of immature lymphocytes into CD8 T cells (213). Prolonged viral infections resulted in decreased expression of IL-7Rα, which in turn resulted in decreased numbers of circulating memory CD8 cells. It had also been observed that chronic infection with lymphocytic choriomeningitis virus (LCMV) resulted in poor CD8 T cell responses to cytokine stimulation and poor development of a memory CD8 response (214). A similar effect had been observed in a large number of mouse studies, suggesting that chronic viral infections could impair many aspects of CD8 effector T cell function (215–218).

Chronic viral infections have been found to have differential abilities to induce an immune suppressive state depending on the strain of virus. In a mouse model, high levels of T cell exhaustion have been induced by infections with LCMV, hepatitis B virus, hepatitis C virus, and HIV (217–221). Less pronounced immune exhaustion was produced in mouse CD8 cells by infection with Epstein-Barr virus (EBV), measles virus, and CMV (222–225). Why different chronic infections produce differing degrees of immune suppression is not well-understood, but could be due to novel immune evasion mechanisms, as in the case of HIV (220), the rate and degree of replication of a virus, its preferred replicative niche or its degree of interaction with PRRs. One well-characterised example of virally induced immunosuppression can be found in measles virus (225). Measles virus has several ways in which it can interact with the immune system and suppress responses. One of these is by interaction with the CD150 receptor to increase the apoptosis of immune effector cells while also decreasing expression of IFN-γ (226). Infection with measles virus has also been shown to decrease the proliferation of lymphocytes for several weeks after the initial infection (227). One study found that measles virus infection may have immunosuppressive effects on the host immune system that can persist for 2–3 years (228). Although the mechanism by which this may occur was not characterised, it was hypothesised that measles virus employs a method of depleting memory B and T cells, resulting in a heightened susceptibility to infection by other pathogens (229). This process is multifactorial and not fully understood. One proposed mechanism is the inhibition of T cell replication by the measles virus proteins H and F_1_-F_2_ (230). It is possible that other viruses may utilise other immune evasion mechanisms that may result in immunosuppression.

Viral reactivation is a common feature of all forms of sepsis. As excessive inflammation gives way to immune exhaustion some latent infections can take advantage of this more forgiving environment, escape immunological control and replicate. Herpesviruses are most commonly associated with this phenomenon, owing to their ability to become latent for many years (231). Epidemiological studies have found CMV, EBV and HSV to be latent in around 50.4% (232), 66.5% (233), and 53.9% (234) of the non-paediatric population of the United States, respectively, with detection of viral RNA (indicating active replication) in cases of sepsis occurring in 24.2, 53.2, and 14.1% of cases, respectively (235).

Some reactivated viral infections are particularly associated with increased mortality during sepsis. While viral reactivation is associated with higher mortality during sepsis, the contribution of individual viruses to mortality is still controversial. CMV reactivation has been associated with the doubling of mortality rates, which is comparable to the increase in mortality rate associated with latent HIV infection during sepsis (236). This model has been questioned however, and it is possible that the increased mortality attributed to CMV reactivation could be due to the reactivation of multiple viruses simultaneously. One study found no single virus to be significantly associated with higher mortality upon reactivation in sepsis, but found the reactivation of multiple viruses at once to significantly associate with worsened outcome (237). Further research on the contributions of the reactivation of individual virus strains to mortality is required.

It has also been hypothesised that viral reactivation may contribute to a feedback loop within sepsis, wherein the reactivated viruses contribute even further to T cell exhaustion and immunosuppression, resulting in even greater susceptibility to viremia, compounded immune suppression (235) and elevated inflammation. This feedback loop would explain why a high degree of viral reactivation (of all viruses) correlates with increased mortality. It is still unclear whether elevated viral loads of herpesviruses following reactivation indeed impairs lymphocyte function or whether it is just a side effect of other kinds of immune suppression already taking place (238). It has been demonstrated that CMV infection can have an effect on the differentiation of immature lymphocytes into effector CD8 T cells; however, the overall effects this may have on the patient are not well-characterised (239, 240). The state of CD8 differentiation brought about by CMV infection is similar to the differentiation state of CD8 T cells in older adults. This suggests that CMV infection may contribute to immunosuppression, but more research is required to confirm if this effect has a significant effect on mortality during severe infections (240). Immunosuppressive effects have also been observed in EBV infection. EBV infection was found to be associated with reduced antibody responses to vaccines in Gambian infants (241). The mechanism of this is still not yet known. Interestingly CMV infection was not found to be associated with these lowered responses (241). In addition, there is evidence that these reactivated viral infections contribute to inflammation alongside any immunosuppressive effects, resulting in a heightened state of inflammation but without the capacity to resolve it. In particular, HSV and CMV have been associated with inflammatory responses upon reactivation (242, 243). Whether or not this phenomenon contributes significantly to mortality during sepsis is still controversial (235).

Understanding the role viral reactivation plays in the immunosuppressive phase and in the pathology of sepsis may provide avenues to treatment in the future. Possible approaches would be the application of antiviral medications specific to viruses that commonly experience reactivation, or drugs that could prevent immune exhaustion or ameliorate its effects (like inhibitors of negative co-stimulatory molecules discussed below). An improved understanding of the effects of viral reactivation is vital to expanding our understanding of sepsis and will contribute to better categorisation of illness and application of more appropriate treatments.

## Is viral sepsis different from bacterial sepsis?

The diagnosis of viral sepsis can be useful to inform treatment in cases where antiviral medications are available and suitable; however, immunological data are scarce on viral sepsis. It cannot be said with any certainty if viral sepsis is meaningfully different from bacterial sepsis. Viral sepsis is only understood insofar as immune responses involved in severe viral infections are understood. The causes and character of sepsis can be highly heterogeneous (206). While knowledge of the causative pathogen provides with treatment options against that pathogen and against associated immune responses, sepsis and subtypes thereof are not characterised well enough for immune response-based therapies to proliferate and enter the mainstream. An important next step in the understanding of sepsis will be the characterisation and grouping of sepsis cases according to some criteria that may inform treatment, and the discovery of cheap and effective biomarkers which would allow these criteria to be defined.

Studies have been conducted which have aimed to develop methods to discriminate between viral and bacterial infections (244–246). Some of these have analysed transcriptomics to identify gene signatures that can differentiate between viral and bacterial infections (244–246). Some genes identified in these studies include genes downstream of the IFN signalling pathways such as IFN-stimulated gene 15 (245) and IFN-α-inducible protein 27 (244) as well as cytokines such as IL-16 (245). The roles these genes play in viral infection is not yet known; however, that information is not required for their use as biomarkers, and as such they may have clinical utility regardless. The results of these studies may inform future research to identify biomarkers which can be used in a clinical setting to quickly differentiate between bacterial and viral infections. Applications of these methods to cases of sepsis may help us develop an understanding of how sepsis differs for differing aetiologies. Transcriptomic studies in sepsis have been performed before as discussed above (201, 202), but more focused studies aiming to understand the pathogen's role in the character of the disease will be essential to future sepsis research and providing an answer to the mysteries of viral sepsis. It may prove difficult to recruit sufficient sample sizes for a highly powered transcriptomics study in viral sepsis due to its underdiagnosis.

## Treatments for viral infections and sepsis

Up to 42% of all cases of sepsis are culture negative, suggesting a possible non-bacterial cause of infection (21), if appropriate tests have been performed. Despite this, however, the preferred treatment of sepsis in all cases is the early administration of broad-spectrum antibiotics. The survival rate of patients presenting with septic shock decreases by an average of 7.6% for every hour that antimicrobials are not applied (247), with time to application of antimicrobial therapy being the single greatest indicator of outcome in the multivariate analysis performed in one study (247). However, the administration of antibiotics will not be effective in the case of viral sepsis and can be associated with adverse effects. Understanding a potential viral cause of the disease increases the possible treatment options, opening the possibility of using broad-spectrum antiviral medications, but also to treatments built on an understanding of both sepsis, and how the immune response to pathogens may contribute to it.

Prospective treatments for specific viruses implicated in sepsis are being developed. Pleconaril is an antiviral against enteroviral infection which inhibits viral attachment to the hosts cell receptors and prevents uncoating of the viral nucleic acids. There are data suggesting that the drug would be effective and safe in neonatal virally induced sepsis (248). The drug recently completed a small phase 2 clinical trial (248); however, it is no longer under development and is not available, even for compassionate use. The benefits of more effective antiviral medicines are clear. They could help both in cases of severe viral infections and sepsis while also providing treatment for more benign infections. It could prove useful to use such drugs alongside antibiotics in sepsis cases, to allow for the possibility of a viral cause, although this would also open the possibility of the development of viral resistance. There are many specific antiviral drugs that have been developed and gone through trials; however, none have yet been tested specifically for sepsis. Examples that may be beneficial in presentations of sepsis in certain situations include acyclovir, which has been proven effective in HSV infections (249), amantadine, rimantadine, oseltamivir, and zanamivir for influenza (250, 251), and more broad-spectrum antiviral drugs like ribavirin and favipiravir (250).

Antiviral medicines may also have a role in the treatment of viral reactivation, which may improve outcomes even in non-virally induced sepsis. Ganciclovir has been demonstrated to measurably decrease CMV reactivation in mice (248); however, human trials did not show any significant decrease in CMV-induced inflammatory cytokine levels (252). There have been numerous other drugs developed against CMV (253) which could be effective in preventing viral reactivation.

With the advent of research into personalised medicine, the idea of treating the host immune response in sepsis has become popular. By understanding the host response to pathogens and modifying it we may prevent serious infections that can result in sepsis or sepsis-like-illness. One common strategy of treating the host response is the use of immunomodulatory molecules to prevent harmful excessive inflammation in infections. Immunomodulation in sepsis aims to decrease the harmful effects of excessive inflammation by altering or counteracting the effects of inflammatory mediator molecules (254), such as TNF-α (255) or by using broad anti-inflammatory molecules, such as corticosteroids (256). Most trials for immunomodulatory drugs, however, have failed (257). This approach, has fallen out of favour in recent years as it either proves ineffective [e.g., dengue (258, 259)] or in some cases has the opposite of the desired effect and dampen the immune system in such a way as to allow the pathogen freedom to replicate and proliferate into an uncontrolled infection that causes greater harm [e.g., corticosteroids in influenza (260, 261)]. However, adjunctive corticosteroid therapy may be beneficial and can be considered in patients with varicella zoster virus encephalitis (262, 263) or HSV encephalitis (264).

In recent years, an immunostimulatory approach to immunotherapy against sepsis has become much more popular (265). This approach aims to promote rapid pathogen clearance, decreasing the chance for it to proliferate and cause a more severe infection (265). One proposed method of doing this is selective application of immunostimulatory cytokines such as IL-7 and granulocyte-macrophage colony-stimulating factor (GM-CSF) which some studies have shown to contribute to more effective viral clearance (266, 267). A recent study demonstrated that the majority of deaths which occur due to sepsis occur on the third day or later (203), after immunosuppression has taken hold, suggests an immunostimulatory approach may prove to be beneficial.

In order for the treatment of the immune response to work as a meaningful way to decrease overall mortality we must better understand how the immune response to infections contributes to the development of sepsis. To this end, the development of biomarkers that could determine the likelihood of an infection becoming harmful due to a lack of ability to clear an infection, or indeed whether the initial immune response will be excessive and harmful, would make these relatively brute force treatments much more effective by timing and targeting their application. An understanding of the excessive inflammatory response to pathogens will allow us to better categorise sepsis states, and group them according to treatability. Some strides forward have been made in this field. A 2016 study (201) identified two distinct host response signatures to sepsis by unsupervised hierarchical clustering, one of which significantly associated with higher 14-day mortality. While this study only analysed bacterial sepsis, the methodology would be extremely useful in expanding our understanding of viral sepsis. The identified signature was characterised by relative immunosuppression as well as increased tolerance to endotoxins, T lymphocyte exhaustion and metabolic dysfunction. It was hypothesised that these individuals experienced more severe disease due to an inability to control infections, resulting in increased pathogen replication. These individuals would also be more susceptible to secondary infection. This study, however, only examined sepsis in adult patients with community acquired pneumonia, which is not necessarily informative of paediatric populations with immature immune systems, other immunocompromised groups or viral infections. A similar study focusing primarily on viral sepsis would be invaluable to the understanding of viral sepsis and would open numerous new avenues for prospective treatments. To be effective in a clinical setting this approach will require the development of rapid transcriptomic analysis methods to be clinically useful in a case of a patient acutely presenting with sepsis. Another study aiming to identify a predictive genomic signature had similar results (202), grouping cases of sepsis using a hierarchical clustering method into two distinct subgroups, one of which correlated with a higher rate of mortality, and was characterised by a more immunosuppressed phenotype. Genes involved in the function of lymphocytes were suppressed, despite the relatively high lymphocyte counts in patients in this group. This study had similar limitations to the other study mentioned above.

This knowledge could potentially be used to inform treatment and the development of immunotherapeutics. It was suggested that patients presenting with this immunosuppressed gene expression signature may benefit from drugs that modulate aspects of the immune response, for example treatment with various cytokines (such as IFN-γ, IL-7, or IL-15) or blockade of receptors that can induce cell death in T lymphocytes (201), such as programmed death 1 (PD-1).

The PD-1 ligand is a promising target for the treatment of viral sepsis. PD-1 has been implicated in the development of the immunosuppressive phase of sepsis by inducing the apoptosis of effector T cells. Continued elevation of PD-1 expression in septic patients has been found to correlate highly with patient mortality (238). PD-1 has been implicated in the pathogenesis of highly pathogenic influenza infections. In more severe Influenza infections PD-1 was expressed at a higher level, while blocking it led to increased CD8+ numbers and reduced viral titres *in vivo* (268). Blocking of PD-1 may allow for vastly improved clearance of serious viral infections, preventing the patient from becoming septic. PD-1 blockade treatment may also be useful in the immunosuppressive phase of sepsis by maintaining the competence of the immune system to clear secondary infections and thus decreasing overall mortality (269–271).

One common feature of sepsis that has emerged as a promising target for treatments is the dysregulation of endothelial barriers (272). The endothelial barrier is a continuous layer coating the vascular system which separates the fluid from the tissue compartments. The barrier is important in maintaining bodily homeostasis, regulating the passage of gases, liquids, proteins, cells and micro-organisms, among other things from the blood into the tissues. This dysregulation has been found to be central to the pathology of sepsis (273). In states of septic shock the tight junctions between cells become disrupted due to platelets and neutrophils adhering to the endothelial wall, the release of inflammatory and toxic mediators by these cells, and an increased expression of binding molecules like selectins and integrins, which allow leucocytes to bind to the endothelial layer, and then migrate through it (274). The process of dysregulation also compounds any damage to the endothelial barrier by increasing the level of inflammatory mediators at its surface. This leads to fluid leaking into interstitial tissues, and the recruitment of macrophages and other inflammatory cells to tissues they cannot normally access resulting in tissue damage (272). While these responses generally allow immune effector cells to reach sites of localised infection or damage, during the dysregulation and move away from homeostasis that occurs in sepsis, they can allow for considerable damage to be done to tissues.

This phenomenon has been found to also occur in serious viral infections, and also plays a major role in the pathology of viral sepsis in these cases (275). Viruses such as hantavirus, dengue viruses, and HSV have presentations that suggest a role for the endothelial barrier in the pathogenesis of serious disease (276). There is a lack of data on the relevance of endothelial leaking in most other viral infections; however, there is evidence that it is involved in the pathogenesis of avian influenza A (H5N1) virus, which produces a cytokine storm effect (277), and is known to increase vascular permeability and immune cell infiltration into the tissues.

Therefore, endothelial barrier dysfunction presents an attractive target for the treatment of severe viral infections and sepsis. There are several drugs which are known to help preserve endothelial integrity which may be of use in treating severe viral infections or sepsis. These include common medications such as statins and angiotensin receptor blockers (278), which have proven benefits in both sepsis, influenza and other critical illnesses. They are thought to work by maintaining or restoring endothelial barrier integrity (278) and could be promising treatments of viral infections known to disrupt endothelial integrity. These drugs have had some success in treating severe viral infections such as Ebola and influenza (279–281) suggesting their use in viral sepsis is a promising area for future research. One trial aimed to control the severity of sepsis using the drug “atorvastatin.” The trial, while aborted due to subpar recruitment, demonstrated interesting results. It was calculated that assuming the drug would bring about a 15% reduction in cases of progression to sepsis, 414 patients would be required to achieve statistical significance. While only a quarter of this number were recruited, an 83% decrease in progression to sepsis (from all causes) was observed, far exceeding expectations (282). However, due to the insufficient sample size, the result was insignificant (282). With the addition of more data these results would suggest that statins may be a particularly promising route for further research particularly in cases of viruses known to interfere with endothelial barrier function.

## Conclusions

Viral sepsis is a continually underdiagnosed and heterogeneous form of sepsis that can be caused by a wide variety of viruses. The most common of these pathogens are HSV, enteroviruses, HPeVs, influenza, and dengue viruses. Some populations are at a much higher risk of viral sepsis than others for many reasons. The populations at the highest risk are young children, pregnant women, older adults, and immunosuppressed individuals. This heightened risk and severity is due to relative immunosuppression present in these populations. The viruses that most often cause viral sepsis tend to have the capacity to evade killing by the immune system while still inducing powerful inflammatory responses, often characterised by high levels of TNF-α and IL-6 expression alongside low IFN-γ expression that can damage the host. The prolonged inflammation that can be brought on by these infections can then result in an immunosuppressed state, further reducing the body's capacity to clear infections, and drastically increasing the risk of death from the original viral infection, a newly acquired infection or a reactivated infection. Understanding the viral cause of sepsis and the immune responses to common viral infections could lead to improved treatment of sepsis by use of specific antiviral medications. In the future it may be possible to apply immunotherapies built around the understanding of the specifics of viral infections to either aid in viral clearance or reduce harm from viral infections. Sepsis from differing causes seems to differ little in its clinical presentation; however, use of modern transcriptomic methods is demonstrating that there are meaningful differences in immune responses that may be used to distinguish between viral and bacterial sepsis, which may aid in the development of future immunomodulatory drugs.

## Author contributions

AP, G-LL, and JM conceived the topic and scope of this review. G-LL and JM drafted the first version of the manuscript. G-LL compiled the table. JM designed the figure. AP and SD provided critical revision of the manuscript. All authors made significant editorial contributions, read and approved the submitted version of the article.

### Conflict of interest statement

AP has previously conducted studies on behalf of Oxford University funded by vaccine manufacturers, but currently does not undertake industry funded clinical trials. AP chairs the UK Department of Health's (DH) Joint Committee on Vaccination and Immunisation (JCVI) and is a member of the World Health Organization's (WHO) Strategic Advisory Group of Experts. The views expressed in this manuscript are those of the authors and do not necessarily reflect the views of the JCVI, the DH, or the WHO. The remaining authors declare that the research was conducted in the absence of any commercial or financial relationships that could be construed as a potential conflict of interest.
